# Association of T-cadherin levels with the response to neoadjuvant chemotherapy in locally advanced breast cancer

**DOI:** 10.18632/oncotarget.14630

**Published:** 2017-01-13

**Authors:** Dedi Kong, Mei-Hong Wang, Jie Yang, Liang Li

**Affiliations:** ^1^ Department of Thyroid and Breast Surgery, Jining No.1 People's Hospital, Jiningy 272011, Shandong, People's Republic of China; ^2^ Department of Pathology, Jining No.1 People's Hospital, Jiningy 272011, Shandong, People's Republic of China; ^3^ Department of Pharmacy, Jining No.1 People's Hospital, Jiningy 272011, Shandong, People's Republic of China

**Keywords:** T-cadherin, neoadjuvant chemotherapy, locally advanced breast cancer

## Abstract

**Purpose:**

To examine the association of T-cadherin with pathologic complete response (pCR) after neoadjuvant chemotherapy for locally advanced breast cancer.

**Results:**

T-cadherin expression before and after neoadjuvant chemotherapy was similar (*P* = 0.162). The multivariable analysis indicated that negative T-cadherin expression was independently associated with pCR after neoadjuvant TAC chemotherapy (*P* = 0.001).

**Materials and Methods:**

A total of 136 patients with locally advanced breast cancer received four cycles of neoadjuvant TAC chemotherapy (docetaxel + epirubicin + cyclophosphamide), followed by surgery. T-cadherin, estrogen receptor (ER), progesterone receptor (PR), HER-2, and Ki-67 were analyzed by immunohistochemistry. The association between T-cadherin expression and pCR after neoadjuvant chemotherapy was analyzed using multivariable logistic analysis.

**Conclusions:**

Negative T-cadherin expression before and after neoadjuvant chemotherapy for locally advanced breast cancer was similar. T-cadherin could be considered an independent factor associated with the efficacy of such therapy.

## INTRODUCTION

Breast cancer is a systemic disease and in its primary stage, the cancer cells can micro-metastasize to other organs via blood or lymph. Locally advanced breast cancer implies Stage III breast cancer and local recurrences [1]. In China, the number of patients with locally advanced breast cancer accounts for 20–30%, which is significantly higher than that in western developed countries [2].

Neoadjuvant chemotherapy (NC) is widely used for locally advanced breast cancer. NC allows the control of the primary tumor, increases the resectability and breast-conserving rates, and decreases the rate of micrometastases, thereby improving long-term survival [3–5]. Indeed, the disease-free survival and overall survival of patients receiving pCR are improved [6, 7].

Nevertheless, there are some problems with the application of NC for breast cancer. Indeed, 20% of the patients with locally advanced breast cancer are not sensitive to chemotherapy and NC will only delay the surgery [8, 9]. Therefore, key issues of NC are how to select the individuals that have the highest probability of pathologic complete response (pCR), how to predict and monitor efficacy, and how to accurately evaluate efficacy.

pCR is the gold standard for evaluating chemotherapeutic effects and can partially predict the prognosis [10, 11]. pCR correlates with immunohistochemical markers of breast cancer and these markers could be used to individualize the therapy. Therefore, many studies have reported factors predicting NC efficacy, including estrogen receptor (ER), progesterone receptor (PR), p53, C-erbB-2 (or HER-2), and Ki-67, among others [12–15], but the sensitivity of these factors is poor. Additional and better markers are necessary to predict the efficacy of NC.

A recent study has indicated that T-cadherin (also known as H-cadherin and cadherin-CDH13) is associated with malignant tumors [16–19]. Down-regulation of T-cadherin is associated with an increased risk of malignancy development [20, 21]. A previous study by our group showed that the occurrence of T-cadherin negativity in locally advanced breast cancer (23.2%) was significantly higher than that in Stage I–II breast cancers (6.0%, *P* = 0.001) [22], but the difference in T-cadherin expression before and after NC in locally advanced breast cancer as well as its potential association with prognosis after NC remain unclear.

Therefore, the present study used immunohistochemistry to analyze T-cadherin expression before and after NC in locally advanced breast cancer samples, and to examine the association of T-cadherin expression with pCR after NC.

## RESULTS

### Characteristics of the patients

All patients were women of 30–67 years of age (mean, 48.3 years). They had measurable tumor foci with diameter ≥ 3 cm as detected by mammography or B-mode ultrasound. The detailed clinical and pathological data are shown in Table 1.

**Table 1:** Associations between clinicopathological parameters before NC with pCR

Parameters	*n*	pCR	non-pCR	*P*-value
Age (years)				
< 50	59	9	50	0.316
≥ 50	77	17	60	
Tumor size (cm)				
< 5	43	10	33	0.404
≥ 5	93	16	77	
Lymph node status				
Positive	123	23	100	0.703
Negative	13	3	10	
Menopausal status				
No	84	14	70	0.356
Yes	52	12	40	
ER				
Positive	92	8	84	0.001
Negative	44	18	26	
PR				
Positive	91	9	82	0.001
Negative	45	17	28	
HER2				
Positive	36	9	27	0.295
Negative	100	17	83	
Ki-67				
Positive	110	25	85	0.028
Negative	26	1	25	
T-cadherin				
Positive	94	7	87	0.001
Negative	42	19	23	

### T-cadherin expression

**Figure 1:**
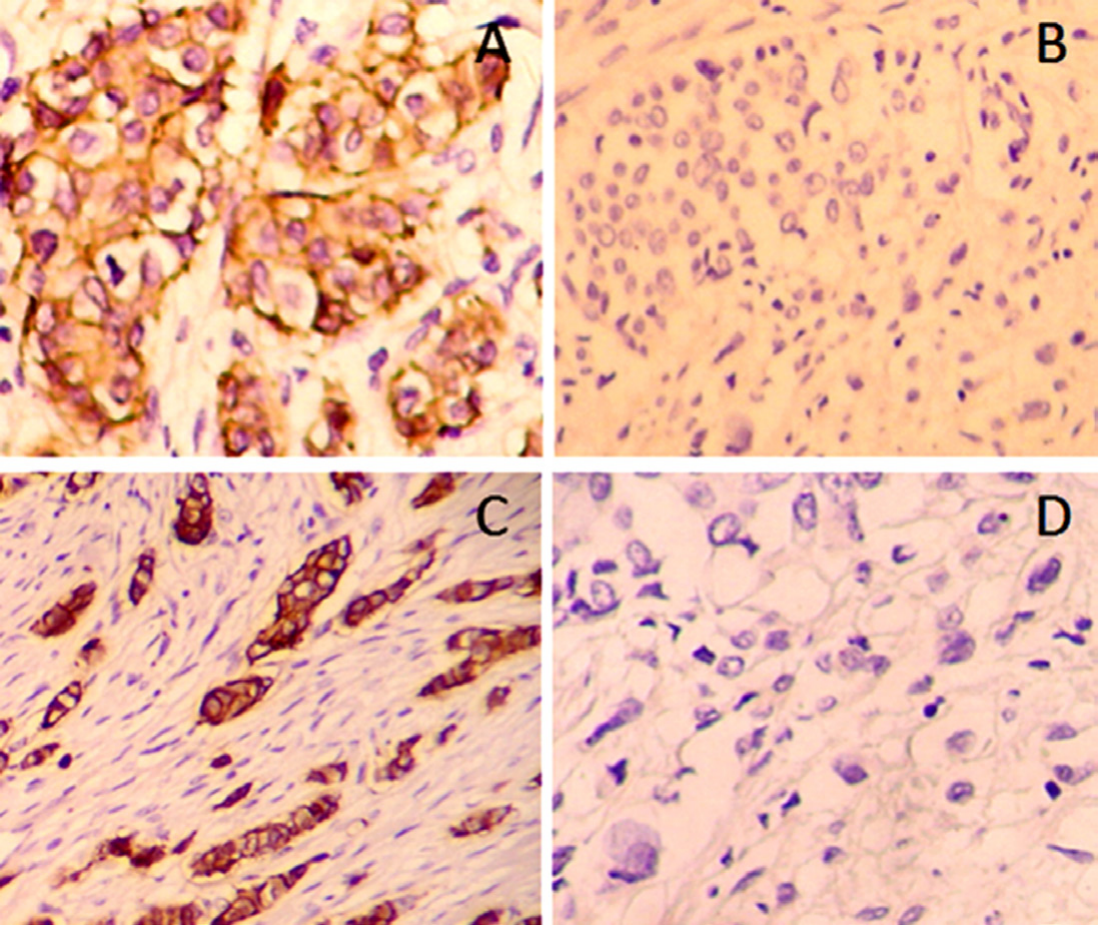
Expression of T-cadherin in histological samples was detected using immunochemistry Images showed representative samples with positive (**A**, **C**) and negative (**B**, **D**) T-Cadherin expression, either before (A, B) or after (C, D) adjuvant chemotherapy (×200).

T-cadherin expression before and after NC was detected by immunohistochemistry (Figure 1). Before NC, there were 92 T-cadherin-positive cases and 44 negative cases. After NC, there were 103 positive cases and 33 negative cases (*P* = 0.162, Wilcoxon rank-sum test) (Table 2). There were no significant differences in ER (*P* = 0.139), PR (*P* = 0.798), and HER-2 (*P* = 0.781) before and after NC. However, Ki67 was significantly decreased after NC (*P* = 0.001) (Table 1).

**Table 2:** T-cadherin expression in locally advanced breast cancer tissues before and after NC

	ER		PR		Her-2		Ki-67		T-cadherin	
	+	–	+	–	+	–	+	–	+	–
Before NC	92	44	91	45	36	100	110	26	94	42
After NC	103	33	89	47	34	102	64	62	83	53
P	0.139		0.798		0.781		0.001		0.162	

### Pathologic response after NC

There were 26 cases of pCR, 80 of PPR, and 30 of PSD according to the pathological efficacy grading standard. The pCR rate was 19.1%.

Univariate analyses showed that pCR was more frequent in patients with negative T-cadherin (*P* = 0.001), negative ER (*P* = 0.001), negative PR (*P* = 0.001), and positive Ki67 (*P* = 0.028) (Table 1). Multivariable logistic regression analysis showed that negative T-cadherin (*P <* 0.001) and negative PR (*P* = 0.006) were independently associated with pCR (*P* = 0.001, *P* = 0.046, *P* = 0.007, and *P* = 0.007, respectively) (Table 3).

**Table 3:** Multivariable analysis of the factors associated with pCR

	B	*P*-value	OR	95%CI	
				Lower	Upper
ER	–1.164	0.081	0.312	0.084	1.154
PR	–2.081	0.006	0.125	0.028	0.549
Ki67	0.729	0.362	2.074	0.433	9.936
T-cadherin	–3.503	0.000	0.030	0.007	0.132

Abbreviations: B, partial regression coefficient; SE, standard error; RR, relative risk; CI, confidence interval.

The ROC analyses showed that ER negativity had 40.0% sensitivity, 24.3% specificity, and 27.2% accuracy for pCR. PR negativity had 36.0% sensitivity, 26.1% specificity, and 27.9% accuracy for pCR. Ki67 positivity had 84.0% sensitivity, 15.3% specificity, and 27.9% accuracy for pCR. Lastly, T-cadherin negativity had 24.0% sensitivity, 20.7% specificity, and 21.3% accuracy for pCR (Table 4).

**Table 4:** ROC analysis of the accuracy of factors associated with pCR

	Sensitivity (%)	Specificity (%)	PPV (%)	NPV (%)	Accuracy (%)
ER	40.00	24.32	10.64	64.29	27.21
PR	36.00	26.13	9.89	64.44	27.94
Ki67	84.00	15.32	18.26	80.95	27.94
T-cadherin	24.00	20.72	6.38	54.76	21.32

## DISCUSSION

A recent study has indicated that T-cadherin is associated with malignant tumors [16–19] such as melanoma [23, 24], ovarian cancer [25], gastric cancer [26, 27], lung cancer, and breast cancer [28–30]. Positive expression of T-cadherin can inhibit cell proliferation and invasion, increase the sensitivity to apoptosis, and decrease tumor growth, suggesting that down-regulation of T-cadherin is associated with an increased risk of cancer [20, 21]. Its high expression can inhibit cell growth and invasion induced by the epidermal growth factor [31]. A previous study by our group showed that the occurrence of T-cadherin negativity in locally advanced breast cancer (23.2%) was significantly higher than that in Stage I–II breast cancers (6.0%, *P* = 0.001) [22].

The association between T-cadherin expression and the efficacy of NC for locally advanced breast cancer NC remains unclear. It has been reported that T-cadherin can be used as an efficacy predictive factor for NC of breast cancer, guiding the application of chemotherapy and avoiding E-cadherin [32]. In the present study, negative T-cadherin expression was associated with a higher rate of pCR. This is supported by a previous study that showed that patients with positive T-cadherin expression had a worse prognosis [31].

Results showed that the expression of Ki67 after NC was significantly decreased, which is supported by a previous study [33]. A previous study showed that ER and HER2 statuses did not change after NC [34], as observed in the present study, but PR status changed after NC [34]. In the present study, PR status did not change after NC, but PR negativity was associated with pCR after NC. Another study showed that both ER and PR statuses changed after NC [35]. Discrepancies among studies could be due to the study populations, NC regimen, tumor stage, and ethnicity.

The present study is not without limitations. The sample size was small and from a single hospital. Only a limited panel of markers were assessed. A number of confounding factors could not be taken into consideration because the data were either not collected or not available, or because of the small sample size. The sensitivity analyses were limited by the small sample size and because each factor was analyzed alone. Algorithms of multiple factors could be explored to improve the predictive power.

## CONCLUSIONS

T-cadherin was independently associated with pCR after NC for locally advanced breast cancer. It has the potential to be used as a marker for predicting the clinical efficacy of NC in these patients. Selection of more specific therapy regimens according to the expression of T-cadherin in locally advanced breast cancer is promising, and T-cadherin could become a new therapy target.

## MATERIALS AND METHODS

### Study subjects

The study subjects were consecutive patients treated at the Department of Breast Surgery, Jining No. 1 People's Hospital between January 2013 and December 2014. Inclusion criteria were: 1) diagnosis of locally advanced breast cancer (Stage III) confirmed by ultrasound-guided core needle biopsy and imaging; 2) available tissue samples from before (biopsy) and after (surgery) NC; 3) no contraindication to chemotherapy and received NC; and 4) no prior history of chemotherapy, radiotherapy, endocrine therapy, or molecular targeting therapy before chemotherapy. The exclusion criteria were: 1) inflammatory breast cancer or complicated with inflammatory breast cancer; 2) distant metastases according to ultrasound, computed tomography (CT), or bone scan; 3) did not undergo surgery; or 4) incomplete medical record. After biopsy, all patients received four cycles of TAC regimen (docetaxel + epirubicin + cyclophosphamide), followed by surgery.

The present study was approved by the ethical committee of the Jining No. 1 People's Hospital. The study was conducted in compliance to the Declaration of Helsinki and local regulations.

### Neoadjuvant chemotherapy and surgery

After hospitalization, the patients signed an informed consent for the chemotherapy and received NC. The received the TAC regimen (4 cycles of 21 days each): epirubicin 70 mg/m^2^ iv on day l; cyclophosphamide 500 mg/m^2^ iv on day l; and docetaxel 75 mg/m^2^ iv on day 2. All patients underwent hepatorenal function, electrocardiography, and echocardiography to examine their tolerance to chemotherapy. Dexamethasone tablets were administered orally for three days before NC. In order to prevent water-sodium retention and allergy, omeprazole and ondansetron were given as support therapy (for stomach protection and anti-nausea). Based on the hemogram results after NC, adequate G-CSF was given to increase the white blood cells.

Breast conserving surgery (local extended resection plus axillary lymph node dissection), modified radical mastectomy, or radical mastectomy were performed according to the tumor size, location, patient's wishes, and other clinical considerations.

### Outcomes

The description and grading were made according to the features described in *The standard of diagnosis and treatment of breast cancer* edited by the Ministry of Health of the People's Republic of China [36, 37]. The pathological remission degree was divided into three grades. 1) No pathological change (pSD): slight tissue reaction, effective chemotherapy but not sensitive. The reaction area was less than one-third of the section with more invasive carcinoma. Besides, cancer cells survived, and the lymphatic metastasis rate was high. 2) Partial pathological remission (pPR): moderate tissue reaction, moderate sensitivity to chemotherapy. The reaction area was about half of the section with invasive carcinoma and lymph node metastasis. 3) pCR: severe tissue reaction, and very sensitive to chemotherapy. Intraductal carcinoma could be observed on sections, but no invasive component, with extremely low lymph node metastasis rate.

### Detection of T-cadherin

Specimens were processed within 15 minutes after surgery. Sections were stained with H&E for histopathological diagnosis. Immunochemistry was performed to detect T-cadherin, ER, PR, HER-2, and Ki-67. All tissues were immediately fixed in 10% formalin and embedded in paraffin. The tissues were sectioned into 20 3–4-μm sections. One was stained by H&E and 12 were used for immunohistochemistry. Mouse anti-human T-cadherin monoclonal antibody, immunohistochemistry SP kits, DAB, and PBS buffer were from Fuzhou Maixin Biotech. Co., Ltd. T-cadherin positive staining was mainly located in the cell membrane. The staining results were divided into four grades by semi-quantitative analysis [22]. Staining strength: 0 (no color), 1 (light yellow), 2 (claybank), and 3 (sepia). Stained cell percentage: 0: < 20%; 1: 21–50%; 2: 51–75%; and 3: >76%. The score was calculated by the product of the stained cells rate and staining strength. The final results were divided into: 0, negative (–); 1–3, weakly positive (+); 4–6, moderately positive (+); and 7–9, strongly positive (+++). For analysis, the results were divided as negative (0–3) and positive (4–12).

ER, PR, HER-2, and Ki-67 were evaluated according to the *Guidelines for immunohistochemical detection of hormone receptors* of the American Society of Clinical Oncology and American Society of Pathologists (ASCO/ASP) and the new standard of biological factors detection recommended by the *St. Gallen International expert consensus on initial treatment for early breast cancer* [38, 39].

### Data collection

All pathological diagnoses were performed by two experienced pathologists. Discrepancies were ruled out by consultation with a third pathologist. Demographic and clinical data were obtained directly from the charts.

### Statistical analysis

SPSS 17.0 (IBM, Armonk, NY, USA) was used to analyze the data. T-cadherin expression before and after NC was analyzed by the Wilcoxon rank-sum test. The association between T-cadherin and efficacy of NC was tested using the chi-square test. Variables associated with pCR in univariable analyses were included in the multivariable logistic regression analysis. Receiver operating characteristic (ROC) curves were used to determine the accuracy of the receptors for pCR. *P <* 0.05 was defined as statistically significant.
